# Social disadvantage, insufficient sleep, and cardiovascular disease

**DOI:** 10.3389/frsle.2025.1500218

**Published:** 2025-03-24

**Authors:** Gerard Anthony Kennedy

**Affiliations:** School of Health & Biomedical Sciences, College of Science, Engineering & Health, RMIT University, Bundoora, VIC, Australia

**Keywords:** insufficient sleep, social disadvantage, cardiovascular disease, insomnia, hypertension, stroke

## Abstract

The aim of this paper was to search the literature examining the relationships between social disadvantage, insufficient sleep, and cardiovascular disease and conduct a brief narrative review. A sleep disparity exists in the population with poor sleep quality strongly associated with poverty. Factors such as ethnicity, annual income, education, employment, and health status significantly mediate the effect in poorer disadvantaged people. In turn, the findings from large epidemiological studies show that insufficient sleep and/or insomnia, and/or short duration sleep are linked to increased risk of cardiovascular diseases such as hypertension, coronary heart disease, heart failure, stroke, arrhythmia, and cardiovascular mortality. In addition, insomnia together with objectively assessed short sleep duration confers a higher risk of developing cardiovascular diseases. However, more large epidemiological studies controlling for obstructive sleep apnoea are needed to fully confirm these findings.

## Introduction

The aim of this paper was to search the literature examining the relationships between social disadvantage, insufficient sleep, and cardiovascular disease and conduct a brief narrative review. Insufficient sleep is term that is used to describe chronic and/or habitually short sleep duration and/or poor sleep quality. This condition intersects with insomnia which is defined as difficulties in the initiation and/or maintenance and/or timing of sleep which may be acute lasting <3-weeks and/or chronic lasting more than 3-weeks or even months or years. Insomnia is the most common sleep disorder and a recent meta-analysis that included 13 large observational studies found that the pooled prevalence of insomnia in the general population was 22.0% [*n* = 22,980; 95% *CI*: 17.0–28.0%] and that females had a significantly higher prevalence of insomnia in comparison with males [Odds Ratio *(OR)*: 1.58; 95% *CI*: 1.35, 1.85, *Z* = 5.63, *p* < 0.0001; Zeng et al., 2020]. Healthy sleep duration in adults has been defined as being in the range of 7–9 hours per night (Hirshkowitz et al., 2015). However, a number of other parameters also determine whether or not sleep quality is good. These factors include sleep continuity, sleep depth, and night to night variability in other sleep parameters and subjective perceptions of whether or not sleep quality is good. Key sleep parameters include sleep latency (time to fall asleep) total sleep time, sleep efficiency (time asleep/time in bed), and wakefulness after sleep onset (number/duration). Sleep parameters are objectively measured using polysomnography (PSG) or if this is not practical actigraphy provides a good estimate of sleep parameters. Objective polysomnographic measurement of sleep provides a record of the orderly or non-orderly (in the case of certain sleep disorders) progression through the stages of sleep in each 90 min cycle (3–5 cycles per night usually) across the total night of sleep. Subjective sleep quality is assessed using validated questionnaires and/or sleep diaries that assess perceptions of satisfaction with sleep and parameters such as sleep latency, sleep maintenance, awakenings, and feeling of refreshment on waking after a night of sleep. The Pittsburgh Sleep Quality Index and the Insomnia Severity Index are two measures that are commonly used. The cost of using PSG in epidemiological studies to assess the relationships between sleep parameters and cardiovascular disease are prohibitive. Therefore, most studies use single item questions inquiring about sleep duration and quality asked at a single time point and thus do not assess sleep behavior over an extended period of time.

Studies have reported that socioeconomic factors are strongly associated with poor sleep habits, insufficient sleep, insomnia, other specific sleep disorders and all cause morbidity and mortality (Ferrie et al., 2007; Heslop et al., 2002; Hublin et al., 2007; Kripke et al., 2002; Patel et al., 2010). Patel et al. (2010) conducted a cross sectional study of 9,714 randomly selected people to explore self-reported sleep quality in relation to primary socioeconomic factors such as poverty, annual income, employment status and educational levels. Secondary factors include variables like, poor diet, lack of exercise, and substance use/abuse. Significant differences were found in the outcome variable (poor sleep quality) for race (African American, and Latino vs. White) with the former two groups showing lower sleep quality. Health indicators significantly negatively influenced sleep quality to a greater degree in poorer people. After adjusting for education, employment, and health indicators the association between income and poor sleep was still evident in poor white people but no longer significant in poor African-Americans. Post-college education protected people against poor sleep regardless of other factors. Patel et al. (2010) concluded that a sleep disparity exists in the population with poor sleep quality strongly associated with ethnicity and poverty. In summary, factors such as ethnicity, education, employment, and health status significantly mediate the effect in poor people which as suggested by Patel et al. (2010) may be due to differential vulnerability to these factors in poor relative to non-poor people in relation to sleep quality. In turn, poor sleep has been linked to increased risk of cardiovascular diseases. The findings from large epidemiological studies show that insufficient sleep and/or insomnia, and/or short duration sleep are linked to increased risk of cardiovascular diseases such as hypertension, coronary heart disease, heart failure, stroke, arrhythmia, and cardiovascular mortality. In addition, large epidemiological studies show that insomnia coupled with short sleep duration is even more strongly associated with risk than either of these conditions are alone (Vgontzas et al., 2010, 2013).

## Hypertension

Large cohort studies have shown that reduced sleep duration and lower sleep efficiency are predictive of elevated systolic and diastolic blood pressure and markedly increase the risk of hypertension (Knutson et al., 2009). A Korean study that followed 1,715 people aged 40–70 years old with no hypertension over a median time of 2.6 years found that people who reported sleep durations of ≤ 6 h showed increased levels of hypertension (*OR*: 1.71%; 95% *CI*: 1.01–2.89) in comparison with people sleeping 6–7.9 hours per night (Yadav et al., 2017). Similarly, a study in the USA of 1,741 men and women found that short sleep duration measured via PSG was strongly associated with elevated blood pressure (Fernandez-Mendoza et al., 2017a). Objectively measured short sleep has also been shown to play a key role in mediating the relationship between hypertension and all-cause mortality (Fernandez-Mendoza et al., 2017b).

A large prospective English study of 3,086 men and women aged 50 years and over showed that self-reported short sleep duration was predictive of hypertension in men and women aged <60 years old but not in older people (Jackowska and Steptoe, 2015). A study of 4,810 people followed for between 8 and 10 years found an interaction between age and sleep duration (Gangwisch et al., 2006). This study showed that 5 or less hours of sleep per night was associated with increased risk of hypertension [Hazards Ratio (*HR)*: 1.51; 95% *CI*: 1.17–1.95] in younger people (32–59 years old) but not in older people. In addition, it was found that a higher percentage of people developing hypertension reported <7 h sleep per night in comparison to those who slept 7–8 hours per night but only in the younger group. Another study has shown an 8% increase in risk for hypertension in a sample of 161,121 men and women free of other diseases including obesity (Deng et al., 2017). In addition, two meta-analyses have shown increased risk of hypertension in people with short sleep duration (Wang et al., 2015; Meng et al., 2013).

Studies of insomnia also support an association between this sleep disorder and hypertension. However, the data from these studies are more variable due to differences between studies in how insomnia was defined and assessed (Javaheri and Redline, 2017). There is also evidence that insomnia together with short duration sleep predicts hypertension more strongly than either disorder does alone. Insomnia present for at least 1 year together with short duration sleep verified via PSG was associated with a greater increase in the odds of hypertension (*OR*: 3.75; 95% *CI*: 1.58–8.95) in comparison to insomnia alone (*OR*: 2.24; 95% *CI*: 1.19–4.19; He et al., 2017).

A cross-sectional study of 255 people with clinically diagnosed insomnia and short sleep (≤ 6 h) verified via PSG found that they had an increased prevalence of hypertension in comparison to those with sleep >6 hours per night (Bathgate et al., 2016). Interestingly, these authors also found that those with subjectively reported insomnia and short sleep did not show hypertension.

Some studies have shown that reduced time in slow wave and rapid-eye-movement sleep stages together with lower sleep efficiency are associated with attenuated blood pressure dipping in otherwise healthy adults (Hinderliter et al., 2013; Loredo et al., 2004; Silva et al., 2000). Similarly, poorer quality of sleep contributes attenuated blood pressure dipping in African Americans in comparison to Caucasian Americans (Sherwood et al., 2011). In addition, reduced sleep efficiency has been shown to be associated with signs of pre-hypertension in adolescents (Javaheri et al., 2008).

More recently, Chaput et al. (2024) examined sleep regularity and major adverse cardiovascular events in a prospective cohort study of 72 269 adults in the UK aged 40–79 years old. Patients wore wrist accelerometers for 7 days and sleep regularity index (SRI) scores were calculated using a validated algorithm, and patients were then categorized as irregular (SRI < 71.6), moderately irregular (SRI between 71.6 and 87.3), and regular (SRI > 87.3 (reference group). Data on MACE and its subtypes (myocardial infarction, heart failure, stroke) were accessed from inpatient hospital and death records. Data were collected from 72,269 patients with no previous history of MACE and no recorded cardiac events in the first year of the 8 year follow up. Irregular (*HR*: 1.26; 95% *CI*: 1.16–1.37) and moderately irregular sleepers (*HR*: 1.08; 95% *CI*: 1.01–1.70) were at higher risk of MACE compared with regular sleepers. Joint SRI and sleep duration analyses showed that meeting the age-specific sleep duration recommendation offsets MACE risk for moderately irregular sleepers (*HR*: 1.07; 95% *CI*: 0.96–1.18), but not for irregular sleepers (*HR*: 1.19; 95% *CI*: 1.06–1.35). Irregular sleep was associated strongly with higher MACE risk. Adequate sleep duration was not enough to offset adverse cardiac effects in irregular sleepers.

In summary, the available evidence suggests that insufficient sleep, particularly short sleep duration is associated with increased risk of higher blood pressure and the development of hypertension. Short sleep duration together with insomnia appears to confer a greater risk of developing hypertension.

## Heart disease

Large prospective epidemiological studies have shown that there is a significant association between both insufficient and short sleep with increased risk of cardiovascular disease (CVD) in men and women (Lao et al., 2018; Ayas et al., 2003). Smaller studies tend to find increased risk of CVD only in men (Mallon et al., 2002; Meisinger et al., 2007). However, some studies suggest that short sleep duration combined with disturbed sleep better predicts CVD events in comparison to either short sleep duration or insomnia alone (Chandola et al., 2010). A German cohort study of 3,505 and 3,388 middle-aged men and women monitoring CVD showed a significant association between self-reported short sleep ≤ 5 h and myocardial infarction in women (*HR*: 2.98; 95% *CI*: 1.48–6.03) but not in men. However, there was no association found between self-reported difficulty initiating sleep or difficulty maintaining sleep and myocardial infarctions. A Swedish cohort study of 1,870 men and women aged 45–65 found that poor sleep quality was associated with adverse cardiovascular outcomes in men but not in women. Short sleep duration was not associated CVD in either sex. In terms of poor sleep, difficulty initiating sleep, a key symptom of insomnia, was associated with increased CVD mortality in men but not in women [Relative Risk *(RR)*: 3.1; 95% *CI*: 1.5–6.3; Mallon et al., 2002].

Cohort studies with large numbers of people show that short sleep duration is positively associated with CVD risk. A prospective study of 71,617 women (Nurses) aged 45–65 years old found that self-reported sleep duration ≤ 5 h predicted CVD in comparison to 6 and >7 h sleep duration (*RR*: 1.45; 95% *CI*: 1.1–1.92; Ayas et al., 2003).

A large prospective study in England examined the differences between men and women in terms of insufficient sleep and also examined the relative contributions of sleep duration and sleep quality (Chandola et al., 2010). The study included 10, 308 men and women followed for an average of 15 years to track CVD events including fatalities, myocardial infarctions and angina. There were no sex differences found for sleep complaints and risk of CVD. In the combined sample self-reported disturbed sleep increased the risk of CVD (*HR*: 1.23; 95% *CI*: 1.07–1.43). There was an interaction between self-reported short sleep duration and disturbed sleep. People with short sleep duration ≤ 6 h and poor sleep quality showed the highest hazard ratio for CVD events (*HR*: 1.45; 95% *CI*: 1.24–1.7; Chandola et al., 2010).

A large prospective study of 60,586 Asian adults found that ≤ 6 h self-reported sleep (*HR*: 1.13; 95% *CI*: 1.04–1.23) or difficulty falling asleep/use of sleeping pills (*HR*: 1.31; 95% *CI*: 1.16–1.47) were associated with increased CVD risk (Lao et al., 2018). The interactive effects of both these sleep issues were not assessed, CVD events were self-reported and obstructive sleep anpoea was not controlled for all of which weaken the findings.

Studies have examined the association between insomnia and risk of CVD (Javaheri and Redline, 2017). A study examined subjective insomnia symptoms that negatively affected performance at work in 52,610 men and women with follow-ups over 11.4 years for the first myocardial infarction (Laugsand et al., 2011). It was found that difficulty initiating sleep, difficulty maintaining sleep and complaints of non-restorative sleep were all associated with increased risk of acute myocardial infarction in men and women but that difficulty initiating sleep showed the strongest association with myocardial infarctions (Laugsand et al., 2011). A study in Taiwan matched (*n* = 44,080) people with and without insomnia by age, sex, and comorbidities and followed them for 10 years (Hsu et al., 2015). The study found people with insomnia had a 68% increased risk of developing myocardial infarctions.

Kadier et al. (2022) examined 7,850 participants selected from National Health and Nutrition Examination Survey (NHANES) 2005–2008 aged over 20 years. Using multivariate regression analyses they showed that sleep problems were associated with increased risk of 75% for CVD (*OR*: 1.75; 95% *CI*: 1.41, 2.16). Daytime sleepiness was associated an increased risk of 54% for CVD (*OR*: 1.54; 95% *CI*: 1.25, 1.89). Insufficient sleep showed a 1.42-fold higher likelihood of CVD (*OR*: 1.42; 95% *CI*: 1.13, 1.78). Prolonged sleep-onset latency was associated with an increased risk for CVD (*OR*: 1.59; 95% *CI*: 1.17, 2.15). The association of sleep problems with CVD risk was more pronounced in the group younger than 60 years (*p* for interaction = 0.019), and the relationship between short sleep-onset latency and total CVD differed by sex (*p* for interaction = 0.049) with men affected more.

In summary, large prospective studies report associations between both reduced sleep duration and sleep quality, and the risk of CVD (Lao et al., 2018; Ayas et al., 2003). There is evidence that short sleep duration and poor sleep quality together identifies people at increased risk of CVD (Chandola et al., 2010). Studies of insomnia alone also show associations of this sleep issue and CVD. Short sleep duration combined with insomnia is also a strong predictor of adverse cardiovascular outcomes events (Fernandez-Mendoza et al., 2012).

## Heart failure

Heart failure can cause insufficient sleep due to symptoms such a paroxysmal nocturnal dyspnoea and/or Cheyne Stoke Respiration, use of medications that can cause sleep disruptions (e.g., diuretics) and finally anxiety and depression related to the disease itself. Therefore, there is a bidirectional relationship between sleep and heart failure.

An English study examined the relationships between self-reported sleep duration and heart failure by following 3,723 older men with and without pre-existing CVD but without heart failure for 9 years (Wannamethee et al., 2016). Self-reported sleep duration of ≤ 6 h increased the risk of heart failure in men with pre-existing CVD. Men without pre-existing cardiovascular disease showed no increased risk of heart failure regardless of sleep duration, but daytime napping was associated with increased risk of heart failure (Wannamethee et al., 2016). Excessive day time sleepiness including napping is a common symptom of OSA. However, obstructive sleep apnoea (OSA) was not assessed in this study and is a common problem in older overweight men and women.

Some studies have shown a relationship between self-reported sleep duration and heart failure (Kim et al., 2013; Westerlund et al., 2013). Large prospective cohort studies have shown associations between self-reported insomnia symptoms (difficulty initiating sleep, maintaining sleep, and non-restorative sleep) and increased risk of heart failure (Laugsand et al., 2014; Ingelsson et al., 2007). One study showed that in middle-aged, overweight men, risk was independent of established risk factors for heart failure (Ingelsson et al., 2007). A more recent study by Kadier et al. (2022) showed congestive heart failure when sleep problems were present (128% *OR*: 2.28; 95% *CI*: 1.69, 3.09). They also found daytime sleepiness was associated increases in risk of 54% for CVD (*OR*: 1.54; 95% *CI*: 1.25, 1.89), Prolonged sleep-onset latency was associated with an increased risk of CHF (*OR*: 2.08; 95% *CI*: 1.33, 3.23). In summary, the findings for insufficient sleep suggest that it plays a role in heart failure, however cardiovascular risks are more evident when there are co-existing sleep symptoms. Short sleep duration seems to have a strong impact on heart failure risk in those with established cardiovascular disease.

## Stroke

Short sleep duration (≤ 6 h) has been associated with increased risk of stroke in a number of studies. A study that followed 23,620 middle-aged men and women over 8 years in Europe found that short sleepers were at significantly increased risk for ischemic and hemorrhagic stroke (von Ruesten et al., 2012). A study of 93,175 postmenopausal women aged 50–70 years followed over a 7.5 year period found a significant association between short sleep duration (≤ 6 h) and ischemic stroke in women without pre-existing cardiovascular disease and/or diabetes (Chen et al., 2008). A study of 25–74 year old men and women over a 14 year period showed that short sleep duration (≤ 5 h) was significantly associated with a 2.3-fold increase in stroke in men. Another study of 5,666 of people aged 45 and over followed them for 3 years and found that short sleep duration (≤ 6 h) was associated with an increase in risk of stroke in normal weight people after adjusting for demographics, stroke risk factors, health behaviors, and diet (*HR*: 4.2; 95% *CI*: 1.62–10.84; Ruiter Petrov et al., 2014). However, some prospective studies have not supported these findings (Leng et al., 2015; Kawachi et al., 2016; He et al., 2017). A prospective study of 9,692 stroke-free people aged 42–81 found that long sleep duration but not short sleep duration was associated higher stroke risk (He et al., 2017). However, long sleep might already be a marker of stroke associated issues such as hypertension and other cardiovascular risk factors. In contrast, other studies have shown that short sleep duration is independently associated with increased stroke risk (Li et al., 2016; Pan et al., 2014). A study in Singapore of 63,257 people aged 45–74 showed that short sleep duration was significantly associated with increased risk of ischemic and non-specified stroke mortality in people with hypertension (Pan et al., 2014).

Insomnia has also been implicated as a risk factor for stroke (Wu et al., 2014). Insomnia symptoms and objectively assessed (PSG) short sleep duration (≤ 5 h) are associated with higher risk of heart-rate variability, hypertension, diabetes, neurocognitive impairment, and mortality in comparison to people with objectively assess longer sleep duration (Vgontzas et al., 2013; Fernandez-Mendoza et al., 2017a,b, 2021). A retrospective Taiwanese study of 21,438 people with clinically diagnosed insomnia and 63,314 aged and sex matched people with no insomnia diagnosis, tracked over 4 years, showed that people with insomnia had a 54% greater risk of stroke. Kadier et al. (2022) in their study found that sleep problems were associated with increases in risk of 78% for stroke (*OR*: 1.78; 95% *CI*: 1.32, 2.40). Daytime sleepiness was associated increases in risk of 60% for stroke (*OR*: 1.60; 95% *CI*: 1.09, 2.36). Short sleep-onset latency was associated with a 36% reduction in stroke risk (*OR*: 0.64; 95% *CI*: 0.45, 0.90).

In summary, a number of large studies have shown sleep problems, daytime sleepiness (possible indicator of OSA) and short sleep duration coexistent with insomnia symptoms increase the risk of stroke in comparison to short sleep duration alone (Westerlund et al., 2013).

## Conclusions

Studies show a clear link between a range of factors used to assess social disparity and poor sleep. In summary, factors such as ethnicity, annual income, education, employment, and health status significantly mediate the effects on sleep in poorer disadvantaged people (Patel et al., 2010). Patel et al. (2010) have suggested that the effects on sleep may be due to differential vulnerability to a range of social factors in poorer people relative non-poor people. In turn, poor sleep has been linked to increased risk of cardiovascular diseases.

The research literature supports a strong association between insufficient sleep defined as short sleep duration, reduced sleep quality and/or insomnia symptoms and cardiovascular disease in particular hypertension, coronary heart disease, and stroke. In addition, insomnia together with objectively assessed short sleep duration confers a higher risk of developing cardiovascular disease. However, more large epidemiological studies are needed to confirm these findings. The mechanisms underlying the pathophysiological of this relationship may include increased sympathetic activity, cortisol dysregulation, increased inflammation and vascular dysfunction resulting from insufficient sleep. For example, Kadier et al. (2023) examined the relationship between sleep-related disorders and the systemic immune-inflammation index (SII) in data from 8,505 people enrolled in 2005–2008 National Health and Nutrition Examination Survey (NHANES) in the US. They found that symptoms of OSA, and daytime sleepiness, were significantly positively associated with the SII. Sleep-related disorders were found to have a stronger association with SII in comparison to the platelet-to-lymphocyte ratio (PLR) and neutrophil-to-lymphocyte ratio (NLR). Multiple linear regression analyses showed that participants with sleep problems (β: 21.421; 95% *CI*: 1.484, 41.358), symptoms of OSA (β: 23.088; 95% *CI*: 0.441, 45.735), and daytime sleepiness (β: 30.320; 95% *CI*: 5.851, 54.789) showed positive associations with higher SII scores. Analysis of other inflammatory markers, the PLR and the NLR, showed that only daytime sleepiness was associated with increased NLR ratio scores (β: 0.081; 95% *CI*: 0.002, 0.159).

Many of the studies reviewed have not controlled for obstructive sleep apnoea which disrupts sleep and plays a role in increasing CVD risk. Therefore, there is a need to control for obstructive sleep apnoea in future large epidemiological studies. Future studies should routinely report the apnoea/hypopnoea index (AHI) for all participants wherever it is available. The AHI score can be used to assess the presence of sleep apnoea and also can be used as covariate in analyses assessing the relationships between sleep problems such insomnia/insufficient sleep and cardiovascular risk.

Most of the older large epidemiological studies have used subjective measures to assess sleep issues because of the difficulties and costs of using objective measures (polysomnography) available at the time studies were conducted. More recent studies have begun to use accelerometers (wrist actigraphy) a quasi-objective measure of sleep. Actigraphy has advantages in that the data be used to more objectively assess sleep and its stages and also it has become much more accessible, cost effective and the algorithms used to assess sleep have improved a lot. Zheng et al. (2024) using longitudinal sleep data from commercial wearable devices linked to electronic health record data from the All of Us Research Program showed that sleep patterns, including sleep stages, duration, and regularity, are associated with chronic disease incidence. They included data from 6,785 participants in their study, 71% were female, 84% self-identified as white and 71% had a college degree; the median age was 50.2 years (interquartile range = 35.7, 61.5) and the median sleep monitoring period was 4.5 years (range = 2.5, 6.5). They found that rapid eye movement sleep and deep sleep were inversely associated with the odds of incident atrial fibrillation and that increased sleep irregularity was associated with increased odds of incident obesity, hyperlipidemia, hypertension, major depressive disorder and generalized anxiety disorder.

Yuan et al. (2024) aimed to establish the accuracy of wrist-worn accelerometers for sleep stage classification and subsequently describe the association between sleep duration and efficiency (proportion of total time asleep when in bed) with mortality outcomes. They developed a self-supervised deep neural network for sleep stage classification using concurrent laboratory-based polysomnography and accelerometry. They used 1,113 participant nights of data for training the model. The difference between polysomnography and the model classifications on the external validation was 48.2 min [95% *CI*: for limits of agreement (LoA): −50.3 to 146.8 min] for total sleep duration, −17.1 min for REM duration (95% *CI*: LoA: −56.7 to 91.0 min) and 31.1 min (95% *CI*: LoA: −67.3 to 129.5 min) for NREM duration. The sleep classifier was used in the UK Biobank with ~100,000 participants to examine the association of sleep duration and sleep efficiency with all-cause mortality. In 66,262 UK Biobank participants, 1,644 mortality events were observed. Short sleepers (< 6 h) had a higher risk of mortality compared to participants with normal sleep duration 6–7.9 h, regardless of whether they had low sleep efficiency (*HR*: 1.36; 95% *CI*: 1.18–1.58) or high sleep efficiency (*HR*: 1.29; 95% *CI*: 1.04–1.61). They concluded that sleep accelerometer data has a fair to moderate agreement with polysomnography using their model. Their findings suggest that short overnight sleep increases mortality risk irrespective of sleep continuity.

Finally, actigraphy although offering a reasonable estimation of sleep parameters does not appear to the be final solution to measuring sleep parameters in large studies. It may be that other biological parameters such as heart rate (HR) and respiration rate (RR) which are easily measured strong signals could be used in lieu of accelerometers or in tandem with accelerometers particularly in research on the relationships between sleep disturbances and cardiovascular diseases. Research by Tal et al. (2017) has validated a contact-free system designed to achieve maximal comfort during long-term sleep monitoring, together with high monitoring accuracy. They used a contact-free monitoring system (EarlySense, Ltd., Israel), consisting of an under-the-mattress piezoelectric sensor and a smartphone application, to collect vital signs and analyze sleep. Heart rate (HR), respiratory rate (RR), body movement, and calculated sleep-related parameters from the EarlySense (ES) sensor were compared to data simultaneously generated by the gold standard, polysomnography (PSG). Subjects in the sleep laboratory underwent overnight technician-attended full PSG, whereas subjects at home were recorded for 1–3 nights with portable partial PSG devices. Data were compared epoch by epoch. A total of 63 subjects (85 nights) were recorded under a variety of sleep conditions. Compared to PSG, the contact-free system showed similar values for average total sleep time (TST), % wake, % rapid eye movement, and % non-rapid eye movement sleep, with 96.1% and 93.3% accuracy of continuous measurement of HR and RR, respectively. They found a linear correlation between TST measured by the sensor and TST determined by PSG, with a coefficient of 0.98 (*R* = 0.87). Epoch-by-epoch comparison with PSG in the sleep laboratory setting revealed that the system showed sleep detection sensitivity, specificity, and accuracy of 92.5%, 80.4%, and 90.5%, respectively. This system shows good sleep staging capability with improved performance over accelerometer-based apps, and collects additional physiological information on heart rate and respiratory rate which would be beneficial in cardiac research.
